# Whole-Genomic Characterization of Two Different PRRSV-1 Strains Isolated From a Single Pig

**DOI:** 10.1155/tbed/8260067

**Published:** 2025-09-15

**Authors:** Bangjun Gong, Jiahao Shi, Hu Xu, Chao Li, Lirun Xiang, Zhenyang Guo, Jinhao Li, Siyu Zhang, Zixuan Feng, Haonan Kang, Xueli Zhang, Ziyu Song, Qian Wang, Jinmei Peng, Guohui Zhou, Chaoliang Leng, Kuan Zhao, Yan-Dong Tang, Huairan Liu, Tong-Qing An, Xuehui Cai, Zhi-Jun Tian, Hongliang Zhang

**Affiliations:** ^1^State Key Laboratory for Animal Disease Control and Prevention, Harbin Veterinary Research Institute, Chinese Academy of Agricultural Sciences, Harbin 150001, China; ^2^College of Veterinary Medicine, Hebei Agricultural University, Baoding 071000, China; ^3^Henan Provincial Engineering and Technology Center of Animal Disease Diagnosis and Integrated Control, Nanyang Normal University, Nanyang 473061, China

**Keywords:** coinfection, high-throughput sequencing, PRRSV-1, recombination and evolution, whole-genome characteristics

## Abstract

Recent studies have demonstrated that porcine reproductive and respiratory syndrome virus 1 (PRRSV-1) strains isolated from different farms in China have significant genomic heterogeneity, whereas strains from the same farm present a high degree of genomic similarity. In this study, primary alveolar macrophages (PAMs) and high-throughput sequencing technology were used to successfully isolate and characterize two different PRRSV-1 strains (designated TZJ3556-1 and TZJ3556-2) in a single pig from a farm in Shandong Province with a PRRSV-1 outbreak. Phylogenetic analysis based on ORF5 and complete genome sequences revealed that both TZJ3556-1 and TZJ3556-2 belong to the BJEU06-1-like subgroup. Nucleotide (nt) sequence alignment revealed that the pairwise genomic similarity between TZJ3556-1 and TZJ3556-2 was only 86.03%. Additionally, both strains exhibited the characteristic 5 (4+1) discontinuous amino acid (aa) deletion in the Nsp2 region. Recombination analysis revealed that TZJ3556-1 is one of the parental strains for the recombinant strain TZJ3556-2, contributing Nsp1β, partial Nsp2, partial ORF4, and ORF5-3'UTR genes. This study reports, for the first time, coinfection with multiple PRRSV-1 strains in a single pig and the emergence of an intrasubgroup recombinant strain within the BJEU06-1-like subgroup. The genetic diversity of PRRSV-1 strains in China is likely to become increasingly complex, and these strains may have overcome physical barriers between farms. The emergence of complex and diverse PRRSV-1 strains poses a serious challenge for the prevention and control of PRRS in China.

## 1. Introduction

Porcine reproductive and respiratory syndrome virus (PRRSV) has caused considerable economic damage to the global swine industry. PRRSV belongs to the order *Nidovirales* and the family *Arteriviridae* [1]. The complete genome of PRRSV, which is approximately 15 kb in length, consists of 5' and 3' untranslated regions (UTRs) and contains over 10 open reading frames (ORFs) [2–4]. Taxonomically, PRRSV can be classified into two species: *Betaarterivirus suid 1* (PRRSV-1) and *Betaarterivirus suid 2* (PRRSV-2) [5]. The representative strains for these species are the Lelystad virus for PRRSV-1 and ATCC VR-2332 for PRRSV-2 [5]. Research has demonstrated that global PRRSV-2 strains can be classified into 11 distinct lineages, whereas PRRSV-1 strains can be categorized into four different lineages [6, 7]. In China, all PRRSV-1 strains belong to Lineage 1 (Western European Subtype I) and are further classified into seven subgroups [8–10]. Recent studies have demonstrated a significant increase in both the detection frequency and geographic distribution of PRRSV-1 strains from China [8, 11]. The pathogenicity of Chinese PRRSV-1 is relatively low and does not typically result in mortality in swine [12–16]. However, recent studies have indicated that the virulence of this virus is increasing [17–19]. The recombination patterns of Chinese PRRSV-1 are notably intricate, with recombination breakpoints predominantly localized within nonstructural protein regions [11]. These recombinant strains primarily arise from recombination events between BJEU06-1-like and NMEU09-1-like strains [8, 10].

Research has demonstrated that PRRSV-1 strains isolated from different pig farms exhibit considerable genetic diversity, complicating the elucidation of their evolutionary relationships [8]. However, within the same pig farm, PRRSV-1 is highly homogeneous, indicating that all cases likely originated from a single strain [20, 21]. Recently, we isolated and identified two PRRSV-1 strains with low pairwise similarity from a single pig. High-throughput sequencing technology was used to obtain the complete genomes of two distinct PRRSV-1 strains. A comprehensive analysis was conducted to examine the genomic features and genetic evolutionary relationships of these strains, as well as to explore future trends and control strategies for PRRSV-1.

## 2. Materials and Methods

In 2024, a clinical specimen (lung tissue from one swine) tested positive for PRRSV-1 via real-time RT-PCR. Tissue sample processing and RNA extraction were conducted in accordance with previously established protocols [22, 23]. The remaining tissue homogenate was filtered through 0.45 μm filters and subsequently inoculated onto primary alveolar macrophages (PAMs) for viral isolation. The cultures were harvested after 3 days and stored at −80°C as viral stocks [18, 22, 24]. To determine the presence of PRRSV-1 strains in third-passage cultures from PAMs, the cultures were further tested using real-time RT-PCR and indirect immunofluorescence assay (IFA) [18]. The third-passage cultures from PAMs were subsequently subjected to high-throughput sequencing. The complete genomes of strains TZJ3556-1 and TZJ3556-2 were obtained through next-generation sequencing (NGS) using the MGISEQ-200 platform (MGI Tech Co., Ltd.). The sequence data have been deposited in the GenBank database under the accession numbers PQ726420 and PQ726421. To confirm the isolation of two distinct PRRSV-1 strains from a single pig, we designed two sets of specific primers targeting the same genomic regions for TZJ3556-1 (JDA1-F: GGTGCTGTTGGCTTTCATCCATT, JDA1-R: GGCGACGAGGTAGTCAGCAT; PCR product positions: 442-1966) and TZJ3556-2 (JDI2-F: TTGCTGGCTCTCCGCTGTTT, JDI2-R: GCCTTTCCAAGCCCCTTCACAT; PCR product positions: 446-2275). These primers were used to detect both the third-passage PAMs cultures and the original lung tissue. The PCR products were cloned and inserted into the pMD18-T vector. The plasmids were subsequently sent to Comate Bioscience Co., Ltd. (Changchun, China) for sequencing.

To elucidate the evolutionary relationship between the two PRRSV-1 isolates, two phylogenetic trees based on the ORF5 and whole-genome sequences were constructed using IQ-TREE with the TVM+R4+F and GTR+R5+F models, respectively, using 20,000 ultrafast bootstraps and the Shimodaira–Hasegawa-like approximate likelihood ratio test [25–27]. The pairwise nt and aa similarities of each gene among the two isolates (TZJ3556-1 and TZJ3556-2) and representative strains of the seven Chinese PRRSV-1 subgroups were calculated using Clustal Omega [28]. Subsequently, heatmaps illustrating the pairwise similarities were generated utilizing Pandas v.2.0.3 and Seaborn v.0.13.2 [29].

To detect potential recombination events, the sequence alignment was analyzed using seven algorithms (3Seq, GeneConv, MaxChi, Chimera, RDP, SiScan, and BootScan) with default parameters in RDP4 [30]. Recombination events identified by at least three of the seven algorithms were validated using NCBI BLAST and SimPlot v.3.5.1 [31–34]. A similarity plot was subsequently generated using Matplotlib v.3.6.2 to determine the breakpoints, and phylogenetic trees based on different recombinant segments were constructed to confirm the recombination events [33, 34]. To validate the recombinant segments provided by the parental strains, the genomic regions flanking the recombination breakpoints in TZJ3556-1 and TZJ3556-2 were amplified, cloned, and inserted into the pMD18-T vector for sequencing.

## 3. Results and Discussion

### 3.1. Identification, Isolation, and Genomic Sequencing of Coinfected PRRSV-1 Strains

**Figure 1:**
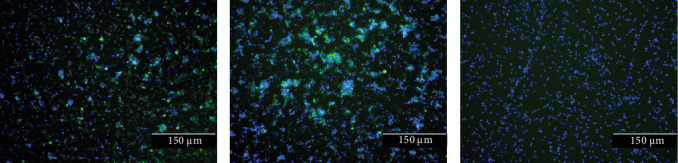
IFA showing the reactivity of a monoclonal antibody against PRRSV-1 to (TZJ3556-1-P3 and TZJ3556-2-P3)-infected (a), ZD-1-infected (b), and control (c) PAM cells. Scale bar = 150 μm.

In May 2024, an outbreak of severe PRRSV-1 infection occurred on an intensive pig farm in Shandong Province. The swine herd in the farm has not been vaccinated against PRRSV. The observed clinical signs of the swine herd were mainly abortion in sows (a miscarriage rate of 8%), and piglets exhibited respiratory distress. A lung tissue sample was collected from an aborted fetus for real-time RT-PCR, virus isolation, and high-throughput sequencing. The real-time RT-PCR test yielded a Ct value of 18.61 for third-passage PAMs cultures. Moreover, the IFA results demonstrated that the PRRSV-1 strains (TZJ3556-1 and TZJ3556-2) were capable of infecting PAMs (Figure 1). These findings collectively indicate that the viruses were successfully isolated from a lung tissue homogenate using PAMs. The third-passage cultures of PAMs were subsequently subjected to high-throughput sequencing. This process yielded complete genomes of two distinct PRRSV-1 strains, designated TZJ3556-1 and TZJ3556-2. The genomes of TZJ3556-1 and TZJ3556-2 were found to be 15,137 nts and 15,103 nt in length, respectively, with a pairwise similarity of 86.03%. To verify that the sampled pig was indeed coinfected with two different PRRSV-1 strains, we used two sets of specific primers (stated above) targeting the same genomic region of TZJ3556-1 and TZJ3556-2 to detect and sequence both the original lung tissue and the third-passage cultures of PAMs. After sequencing, we identified two distinct sequences from the original lung tissue. The pairwise similarity between these sequences was relatively low at 89.3%. Similarly, the same two different sequences were also detected in the third-passage cultures of PAMs. These findings were in agreement with the NGS results and provided further confirmation of the isolation of two distinct PRRSV-1 strains from a single pig. Research has indicated that the whole genomes of PRRSV-1 strains from China that circulate within the same pig farm typically exhibit high similarity [20, 21]. However, in this study, we isolated and identified two PRRSV-1 strains (TZJ3556-1 and TZJ3556-2) from a single pig, and these strains presented relatively low pairwise genomic similarity. Following a comprehensive investigation into the epidemiological history of PRRSV and the breeding management practices at the swine farm, we hypothesize that this phenomenon may be attributed to the movement of personnel, pigs, and semen between this farm and other farms. Furthermore, the appearance of African swine fever has resulted in numerous pig farms in China abandoning the traditional self-breeding and self-rearing model. These farms have transitioned to a model that involves sourcing young pigs from various regions for fattening, thereby lending additional support to the hypothesis stated above. Recently, the genetic diversity of PRRSV-1 within pig farms has undergone significant changes. There is potential for multiple distinct PRRSV-1 strains to coinfect not only the same farm but also individual pigs. These PRRSV-1 strains may have diverged from different ancestral lineages, and recombination events could have occurred among them during evolutionary processes.

### 3.2. Dissecting the Whole Genomes of Coinfected PRRSV-1 Strains

**Figure 2:**
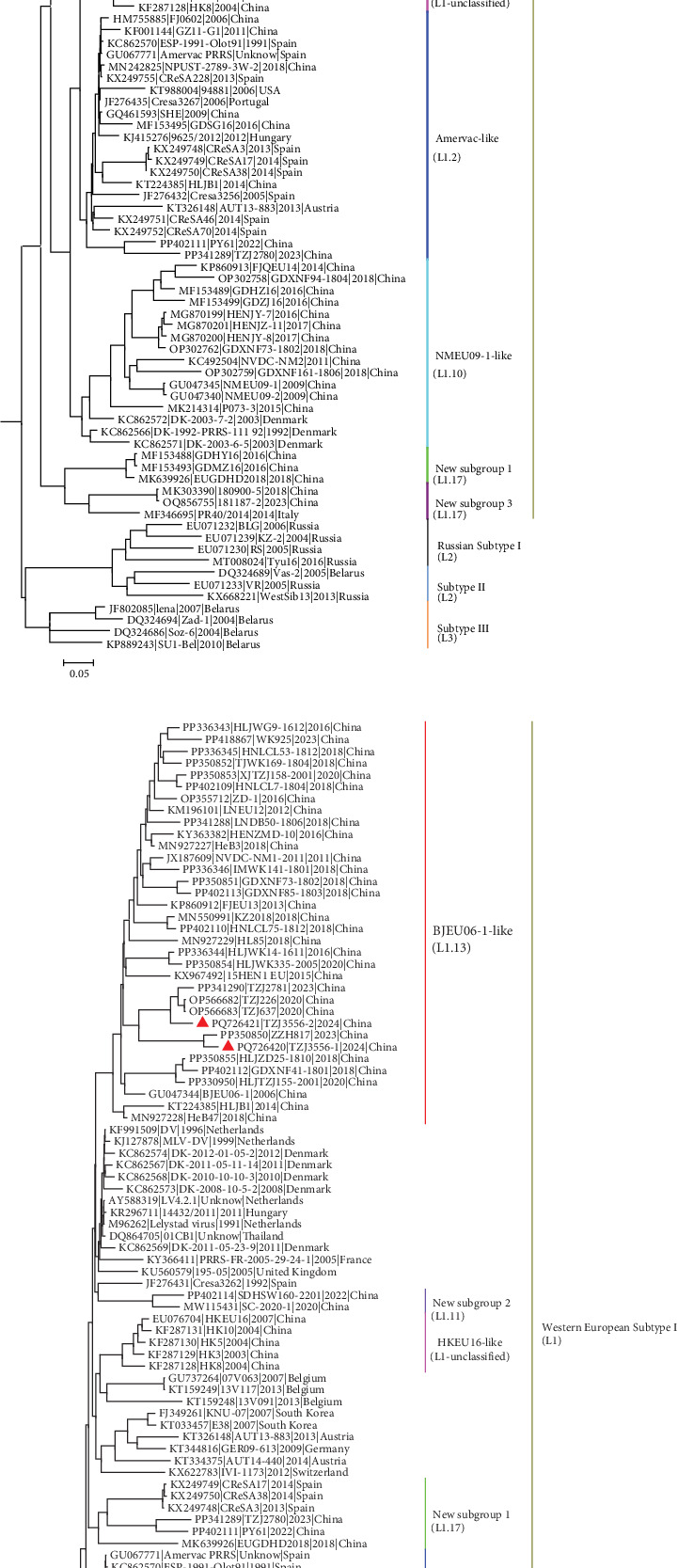
Phylogenetic tree based on the ORF5 (a) and complete genome (b) nucleotide sequences of PRRSV-1 constructed using the maximum likelihood method under the TVM+R4+F and GTR+R5+F models, respectively. The red triangles (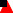) denote the two strains (TZJ3556-1 and TZJ3556-2) analyzed in this study. All PRRSV-1 strains isolated from China belong to Western European Subtype I (L1) and can be classified into seven subgroups. Specifically, TZJ3556-1 and TZJ3556-2 are classified into the BJEU06-1-like subgroup (L1.13).

To elucidate the phylogenetic relationships among TZJ3556-1 and TZJ3556-2, as well as their relationships with various PRRSV-1 strains prevalent in China, we constructed phylogenetic trees based on both the complete genome and the ORF5 gene. The analysis revealed that Chinese PRRSV-1 strains can be classified into seven subgroups. Notably, both TZJ3556-1 and TZJ3556-2 fall within the BJEU06-1-like subgroup (L1.13) in both the whole-genome and ORF5 phylogenetic trees (Figure 2a,b).

**Figure 3:**
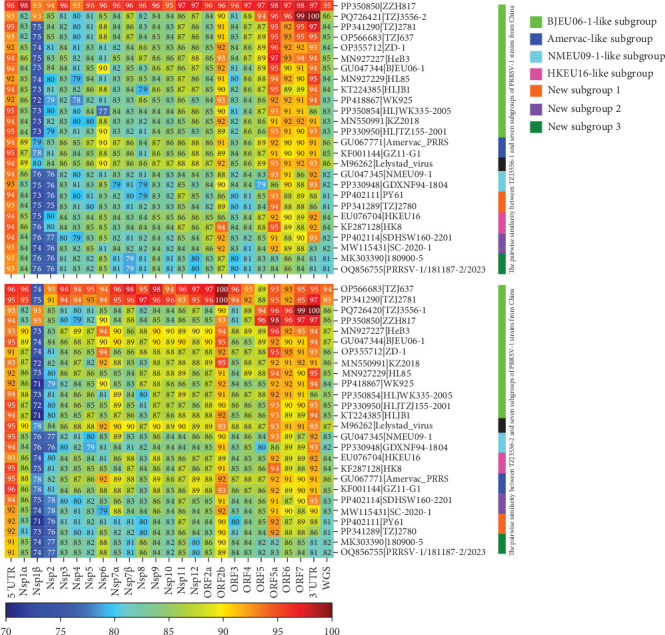
Pairwise nucleotide similarities of each gene between the two isolates, TZJ3556-1 (upper) and TZJ3556-2 (lower), and between the two isolates and representative strains of the seven Chinese PRRSV-1 subgroups. The color bar on the right indicates the seven Chinese PRRSV-1 subgroups, while the color bar at the bottom represents the gradient of similarity. This figure was drawn with seaborn v.0.13.2.

To explore the pairwise similarities of different genes between TZJ3556-1 and TZJ3556-2, as well as their similarities with representative strains of the seven Chinese PRRSV-1 subgroups, we employed Clustal Omega to calculate the nt and aa pairwise similarities. The nt alignment results demonstrated that TZJ3556-1 is highly similar to TZJ3556-2 in the ORF7 and 3'UTR regions but has significant homology with ZZH817 in the remaining genes, as well as the whole genome (Figure 3). TZJ3556-2 displays high similarity to both TZJ3556-1 and ZZH817 (PP350850) in the Nsp1β, ORF5-7, and 3'UTR genes but shares significant homology with TZJ637 (OP566683) and TZJ2781 (PP341290) in the 5'UTR, Nsp1α, Nsp2-Nsp12, ORF2a, ORF2b, and ORF3-4 genes and the whole genome (Figure 3). These findings indicate that TZJ3556-2 is likely a recombinant strain, and TZJ3556-1 may be one of its parental strains. The aa alignment analysis revealed that the Nsp11, Nsp6, ORF5a, M, and N proteins of TZJ3556-1 and TZJ3556-2 are highly similar, with pairwise identities ranging from 96.43% to 100% (Supporting Information 1: Figure S1). In contrast, the remaining proteins show considerable divergence between the two strains, with pairwise similarities varying between 79.23% and 95.00% (Supporting Information 1: Figure S1). Notably, Nsp1β and ORF3/GP3 presented greater nt and aa variability in the nonstructural and structural protein regions, respectively, between the two strains under investigation and the reference strains of the seven subgroups (Figure 3 and Supporting Information 1: Figure S1).

**Figure 4:**
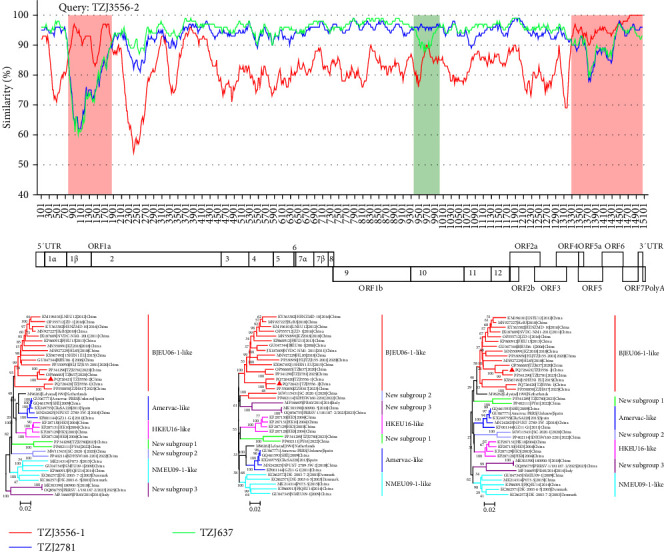
Recombination analysis of the PRRSV-1 strain TZJ3556-2. Sequence similarity comparisons were performed using TZJ3556-2 as the query sequence, with TZJ637 (bright green) as the major parent and TZJ3556-1 (red) and TZJ2781 (blue) as the minor parents. The white background color represents the major parental region, whereas the light red and green background colors represent the minor parental regions. The complete genome structure of PRRS-FR-2005-29-24-1 is shown below the similarity plots, following the phylogenetic tree of the major and minor parental strains with the query strain (TZJ3556-2) marked by red triangles (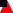). The left phylogenetic tree was inferred from concatenated sequences derived from three genes corresponding to the three white regions in the similarity plot. The middle phylogenetic tree was constructed based on concatenated sequences originated from two genes associated with the two light red regions in the similarity plot. The right phylogenetic tree was constructed using sequences corresponding to the single light green region in the similarity plot.

Recombination events of PRRSV are relatively frequent, and to date, recombination events reported in China include wild-type to wild-type strains, vaccine to vaccine strains, and wild-type to vaccine strains [35–37]. Research has indicated that the recombination events of PRRSV-1 strains from China are mainly intergroup events [8, 10, 11]. To further investigate the relationship between TZJ3556-2 and TZJ3556-1, we initially utilized RDP4 to identify potential recombination events. The results show that TZJ3556-1 is not a recombinant strain, whereas TZJ3556-2 is indeed a recombinant strain (Supporting Information 2: Table S1). Subsequently, the recombination events of TZJ3556-2 were further validated using NCBI BLAST and SimPlot v.3.5.1 and further confirmed by phylogenetic trees constructed based on different recombinant segments. A similarity plot was then generated to precisely determine the breakpoints. The results indicate that TZJ3556-2 is a BJEU06-like intrasubgroup recombinant strain originating from TZJ3556-1, TZJ2781, and TZJ637 (Figure 4). This finding is the first discovery of a BJEU06-1-like intrasubgroup recombination event in China. The major parental strain of TZJ3556-2 is TZJ637, with the minor parental strains being TZJ3556-1 and TZJ2781 (Figure 4). The results of the similarity plot revealed that TZJ3556-2 is a complex recombinant strain, with recombination breakpoints occurring mainly in Nsp1β (nt 773), Nsp2 (nt 1855), Nsp10 (nt 9366 and nt 10011), and ORF4 (nt 13288) (Figure 4). To confirm that the Nsp1β, partial Nsp2, partial ORF4, and ORF5-3'UTR genes of TZJ3556-2 originated from TZJ3556-1, we amplified and sequenced identical regions flanking the recombination breakpoints within the complete genome sequences of TZJ3556-1 and TZJ3556-2 using specific primers (Table 1). Both Sanger sequencing and NGS revealed high similarity between the genes of TZJ3556-2 and those of TZJ3556-1.

Compared to the PRRSV reference strains (Lelystad virus and ATCC VR-2332), regular deletions or insertions in Nsp2 are frequent across different lineages and subgroups. In contrast, mutations between ORF3 and ORF4 are currently not associated with any lineage or subgroup [38–41]. For PRRSV-2, the unique molecular hallmark of NADC30-like PRRSV is a discontinuous deletion of 131 (111+1+19) aa in Nsp2 [42–44]; that of HP-PRRSV is a discontinuous deletion of 30 (1+29) aa [39, 45]; and that of NADC34-like PRRSV is a continuous 100-aa deletion [46, 47]. For PRRSV-1, BJEU06-1-like PRRSV is characterized by a discontinuous deletion of 5 (4+1) aa in Nsp2 [8, 10]. New subgroup 1 PRRSV has 11 continuous aa deletions and one aa insertion in Nsp2 [8, 10]. New subgroup 2 PRRSV has two discontinuous aa deletions (1+1) in Nsp2 [8, 10]. Except for in the case of HKEU16 strain, HKEU16-like PRRSV has a discontinuous deletion of 5 (1+4) aa in Nsp2 [8, 10].

**Figure 5:**
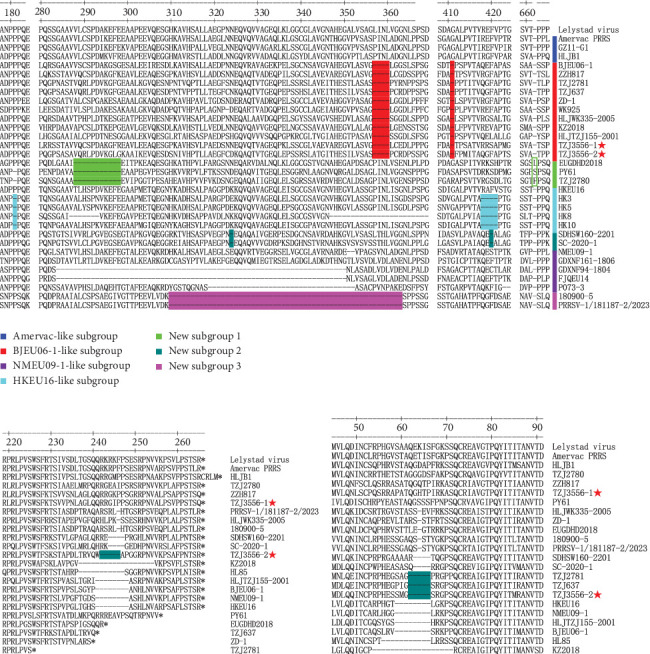
Amino acid alignment of Nsp2, GP3, and GP4 from Chinese PRRSV-1 strains. The red asterisk (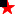) represents the two strains (TZJ3556-1 and TZJ3556-2) identified in this study. (a) Amino acid alignment of partial Nsp2. The seven subgroups (Amervac-like, BJEU06-1-like, NMEU09-1-like, HKEU16-like, New subgroup 1, New subgroup 2, and New subgroup 3) are marked by color bars of deep blue, red, purple, turquoise, bright green, cyan, and pink, respectively. (b) Amino acid alignment of partial GP3. The five continuous amino acid deletion in the C-terminus of GP3 is shown in cyan. (c) Amino acid alignment of partial GP4. The five continuous amino acid deletion in the N-terminus of GP4 is shown in cyan.

To elucidate the aa deletions or insertions in the Nsp2, GP3, and GP4 of TZJ3556-1 and TZJ3556-2, we compared the aa sequences of these three proteins with those of their counterparts originating from representative strains of the seven Chinese PRRSV-1 subgroups and the Lelystad virus. The alignment results for Nsp2 indicated that both TZJ3556-1 and TZJ3556-2 have the classical 5 (4+1) aa deletion at positions 357–360 and position 411 (Figure 5a). The alignment analysis of GP3 and GP4 indicated that TZJ3556-1 has no aa deletions or insertions in these proteins, whereas TZJ3556-2 has a 5-aa deletion in both GP3 (positions 242–246) and GP4 (positions 62–66) (Figure 5b,c).

Phylogenetic analysis and aa alignment revealed that both TZJ3556-1 and TZJ3556-2 are BJEU06-1-like PRRSV and exhibit classical 5 (4+1) discontinuous aa deletions in the Nsp2 gene. These findings confirm that BJEU06-1-like PRRSV has become the predominant circulating strain of PRRSV-1 in China. Previous studies have shown that recombination events of PRRSV-1 strains from China mainly occur between subgroups, and their parental strains have not been found in one pig [8, 10, 11, 35, 37]. This study is the first to identify a BJEU06-1-like intrasubgroup recombinant strain and its parental strains in a single pig, which indicates that the pig farm is concurrently coinfected with multiple PRRSV-1 strains undergoing recombination events within subgroups. These results suggest that the total population size, genomic diversity, and frequency of intrasubgroup recombination events among BJEU06-1-like PRRSV strains may continue to increase in the future.

### 3.3. Application of High-Throughput Sequencing Technology in Veterinary Diagnostics

PRRSV-1 was introduced to China more than 20 years ago [48, 49], and the detection frequency of PRRSV-1 has shown an increasing trend in recent years [8, 11, 36, 50, 51]. Many studies have performed whole-genome sequencing on PRRSV-1-positive samples using conventional RT-PCR and high-throughput sequencing. Notably, until this report, no cases of coinfection with different PRRSV-1 strains had been identified [13, 15, 21, 37, 52–54]. In this study, we employed high-throughput sequencing technology to identify coinfection with two different PRRSV-1 strains in a single pig. This technology could be a powerful tool for the identification of individual viral genomes in complex mixtures and the reconstruction of complete viral genomes of unknown or poorly characterized viruses [55, 56]. Pathogen nt sequences were generated using a suite of bioinformatics software tools to assemble millions of short reads [57]. Thus, using nt sequences, we can discern the profile of pathogenic microorganisms present in a clinical sample. Conventional RT-PCR and real-time RT-PCR, which are limited by the binding affinity of the primer to the template, are primarily used for the differential diagnosis of several specific PRRSV strains [58, 59]. Therefore, when addressing coinfections involving multiple pathogens or different strains of the same pathogen in pig farms, it is crucial to integrate conventional RT-PCR, real-time RT-PCR, and high-throughput sequencing comprehensively for the detection and analysis of various pathogenic microorganisms.

### 3.4. Prevention and Control Strategies for PRRSV-1 Coinfection

The genomic diversity of PRRSV-1 within swine farms has undergone alterations. In the future, multiple strains of PRRSV may coinfect one swine farm or even a single pig, potentially resulting in the emergence of more complex recombinant strains derived from intra- and/or intergroup recombination events. Moreover, the population size and genomic diversity of BJEU06-1-like PRRSV may continue to expand, and the number of recombinant strains derived from BJEU06-1-like PRRSV may gradually increase. The increased genomic diversity of PRRSV-1, coupled with frequent recombination events, poses severe challenges for its prevention and control. The coexistence of different PRRSV strains within a swine farm or a single pig may be attributed to the exchange of personnel, pigs, and semen between different farms. Consequently, when new breeds or semen are introduced, pig farms should strengthen their ability to detect PRRSV from both the antigen and antibody perspectives. PRRS vaccines and antibiotics constitute efficacious interventions for mitigating the incidence of PRRS and managing secondary infections. Vaccines can stimulate protective immune responses against homologous wild-type strains, thereby reducing the viral load of PRRSV in pigs and establishing a robust immune barrier within the herd [10, 60–63]. However, no commercial PRRSV-1 vaccine is currently available in mainland China [36, 54, 64]. Therefore, it is crucial to comprehensively integrate high-throughput sequencing technology to strengthen the epidemiological investigation of PRRSV-1 and to focus on pathogenicity research and vaccine development for the predominant circulating strains.

PRRSV is frequently coinfected with various bacteria and mycoplasmas, including *Haemophilus parasuis* (HPS), *Mycoplasma hyopneumoniae* (Mhp), *Actinobacillus pleuropneumoniae* (APP), and *Pasteurella multocida* (Pm). These coinfections can exacerbate the clinical symptoms observed in pig populations [65–68]. To effectively control secondary infections through the appropriate use of antibiotics, improving the identification and characterization of pathogens coinfected with PRRSV in affected swine farms is imperative.

## 4. Conclusions

For the first time, we report the coinfection of two different PRRSV-1 strains (TZJ3556-1 and TZJ3556-2) in a single pig. The two PRRSV-1 strains exhibit significant genomic divergence, and TZJ3556-1 was identified as one of the parental strains for the recombinant strain TZJ3556-2, which provides direct evidence for PRRSV-1 recombination events. The genomic divergence, recombination events, and coinfection rates of PRRSV-1 strains in China are anticipated to increase in the future, and these strains may have overcome physical barriers between farms, which may present serious challenges for their prevention and control. High-throughput sequencing technology is a powerful tool for the identification of coinfected pathogens.

## Figures and Tables

**Table 1:** Primers for conventional RT-PCR and Sanger sequencing.

Primer names	Sequences	Positions	Target strains
TYP41QS2-F	CGGCACCTTCGTGAGCTACAG	561–2234	TZJ3556-1
TYP41QS2-R	AGCAGTGCTGGACCTTCGAAGTCT		
TYP41CZ5-F	TCGTGAAGCAGCTGCACAGAG	12,565–14,687	TZJ3556-1
TYP41CZ5-R	CTTTATCATCGCGCCCAGCATTTG		
TYP42CZ12-F	TTGCTGGCTCTCCGCTGTTT	446–2570	TZJ3556-2
TYP42CZ12-R	TGCTCGTTCTTCCACGCAAGTC		
TYP42CZ5-F	TCAAACCTTGACGTGGTCACTCATT	12,189–14,672	TZJ3556-2
TYP42CZ5-R	CTTTATCATCGCGCCCAGCATTTG		

## Data Availability

The sequences of this study were deposited in GenBank with the accession numbers PQ726420 and PQ726421. They will be released to the public database when the data or accession numbers appear in print. The sequence data are supplied in supporting files.

## Supporting Information

Supporting Information 1 Figure S1. Pairwise amino acid similarities of each gene between the two isolates, TZJ3556-1 (upper) and TZJ3556-2 (lower), and between the two isolates and representative strains of the seven Chinese PRRSV-1 subgroups. The color bar on the right indicates the seven Chinese PRRSV-1 subgroups, while the color bar at the bottom represents the gradient of similarity. This figure was drawn with seaborn v.0.13.2.

Supporting Information 2 Table S1. Information on recombination events of TZJ3556-2 detected by RDP4 software.
