# Supplementary material for: Whole-Genomic Characterization of Two Different PRRSV-1 Strains Isolated From a Single Pig
Source: Transbound Emerg Dis. 2025 Sep 15;2025:8260067. doi: 10.1155/tbed/8260067 (PMC12453929; doi:10.1155/tbed/8260067)
Supplement: Supporting Information 1 — Figure S1. Pairwise amino acid similarities of each gene between the two isolates, TZJ3556-1 (upper) and TZJ3556-2 (lower), and between the two isolates and representative strains of the seven Chinese PRRSV-1 subgroups. The color bar on the right indicates the seven Chinese PRRSV-1 subgroups, while the color bar at the bottom represents the gradient of similarity. This figure was drawn with seaborn v.0.13.2. [file 8260067.f1.docx]

Fig. S1 Pairwise amino acid similarities of each gene between the two isolates, TZJ3556-1 (upper) and TZJ3556-2 (lower), and between the two isolates and representative strains of the seven Chinese PRRSV-1 subgroups. The color bar on the right indicates the seven Chinese PRRSV-1 subgroups, while the color bar on the bottom represents the gradient of similarity. This figure was drawn with seaborn v.0.13.2.
