# Supplementary material for: Whole-Genomic Characterization of Two Different PRRSV-1 Strains Isolated From a Single Pig
Source: Transbound Emerg Dis. 2025 Sep 15;2025:8260067. doi: 10.1155/tbed/8260067 (PMC12453929; doi:10.1155/tbed/8260067)
Supplement: Supporting Information 2 — Table S1. Information on recombination events of TZJ3556-2 detected by RDP4 software. [file 8260067.f2.docx]

Table S1 Information on recombination events of TZJ3556-2 detected by RDP4 software

|  | Parental Sequence | | Breakpoints | | Detection Methods | | | | | | |
| --- | --- | --- | --- | --- | --- | --- | --- | --- | --- | --- | --- |
| Strains | Minor | Major | Begin | End | RDP | GENECONV | Bootscan | Maxchi | Chimaera | SiSscan | 3Seq |
| TZJ3556-2 | TZJ3556-1 | TZJ637 | 791 | 1848 | 2.08E-78 | 7.93E-50 | 3.50E-77 | 8.49E-26 | 7.44E-28 | 1.97E-30 | 4.44E-16 |
|  |  |  | 13224 | 15107 | 9.31E-53 | 4.38E-18 | 2.68E-36 | 3.57E-19 | 2.80E-20 | 3.39E-21 | 2.22E-15 |
